# Characterization of the Lateral Distribution of Fluorescent Lipid in Binary-Constituent Lipid Monolayers by Principal Component Analysis

**DOI:** 10.1155/2010/125850

**Published:** 2010-04-20

**Authors:** István P. Sugár, Xiuhong Zhai, Ivan A. Boldyrev, Julian G. Molotkovsky, Howard L. Brockman, Rhoderick E. Brown

**Affiliations:** ^1^Department of Neurology and Center for Translational Systems Biology, The Mount Sinai School of Medicine, New York, NY 10029, USA; ^2^Hormel Institute, University of Minnesota, Austin, MN 55912, USA; ^3^Shemyakin-Ovchinnikov Institute of Bioorganic Chemistry, Russian Academy of Sciences, Moscow 117997, Russian Federation, Russia

## Abstract

Lipid lateral organization in binary-constituent monolayers consisting of fluorescent and nonfluorescent lipids has been investigated by acquiring multiple emission spectra during measurement of each force-area isotherm. The emission spectra reflect BODIPY-labeled lipid surface concentration and lateral mixing with different nonfluorescent lipid species. Using principal component analysis (PCA) each spectrum could be approximated as the linear combination of only two principal vectors. One point on a plane could be associated with each spectrum, where the coordinates of the point are the coefficients of the linear combination. Points belonging to the same lipid constituents and experimental conditions form a curve on the plane, where each point belongs to a different mole fraction. The location and shape of the curve reflects the lateral organization of the fluorescent lipid mixed with a specific nonfluorescent lipid. The method provides massive data compression that preserves and emphasizes key information pertaining to lipid distribution in different lipid monolayer phases. Collectively, the capacity of PCA for handling large spectral data sets, the nanoscale resolution afforded by the fluorescence signal, and the inherent versatility of monolayers for characterization of lipid lateral interactions enable significantly enhanced resolution of lipid lateral organizational changes induced by different lipid compositions.

## 1. Introduction

Biomembranes contain a variety of different lipids with a broad array of physicochemical properties. Such lipid variety has stimulated ideas regarding the existence of nonrandom lateral organizational states, including the so-called raft microdomains [1–7]. Model membranes, that is, bilayer vesicles and monolayer films, have proven useful for dissection of the lipid lateral distributional tendencies because simple combinations are possible and adjustments to lipid composition are relatively straightforward. With lipid monolayer systems, imaging of the monolayer surfaces to obtain direct insights into lipid lateral organizational states at macroscopic levels, that is, micron resolution, can be achieved using epifluorescence and/or Brewster angle microscopy [7–10]. These approaches have provided fundamental insights into the role(s) played by line tension and other parameters in stabilizing lipid macrodomains. However, their resolution capabilities are constrained to the micron range, limiting their effectiveness for detection of microdomains.

Recently, we showed that nanoscale changes in lipid packing and lateral organization can be detected in mixed monolayers of 1-palmitoyl-2-oleoyl-*sn*-glycero-3-phosphocholine (POPC) and 1-palmitoyl-2-[7-(4, 4-difluoro-1,3,5,7-tetramethyl-4-bora-3a,4a-diaza-*s*-indacene-8-yl) heptanoyl]-*sn*-glycero-3-phosphocholine (Me_4_BODIPY-PC) by direct monitoring of changes in fluorescence emission from multiple spectra acquired simultaneously with the force-area isotherms [11, 12]. Me_4_BODIPY-PC mimics the behavior of fluid-phase PCs with unsaturated acyl chains and does not perturb lipid packing when the fluorophore concentration is kept low (e.g., ≤1 mole%). At higher mole fractions (e.g., 10 or 20 mole%), the probe begins to exert its own influence on the system rather than serving as a completely “impartial” reporter, a feature observed for virtually all probe molecules. Spectral characterization of Me_4_BODIPY-PC incorporated into lipid monolayers and bilayers indicates retention of excitation and emission transitions associated with monomeric forms of other BODIPY derivatives (i.e., absorption and emission wavelength maxima (*λ*_max _) near 498 nm and 506–515 nm, resp.). Me_4_BODIPY-PC also displays substantial broadening of its emission spectrum as a function of increasing fluorophore concentration due to elevated fluorescence near 570 nm relative to the 510–520 nm region. Although the photophysical mechanism accounting for the emission broadening may differ from the excited state dimer fluorescence of dimethyl-BODIPY derivatives (see Discussion and [11]), the response serves as an effective indicator of Me_4_BODIPY-PC local concentration and lateral distribution within lipid monolayers and bilayers. We recently showed that alterations in Me_4_BODIPY-PC packing density in POPC monolayers, achieved by lateral compression at constant Me_4_BODIPY-PC mole fraction or by variation of Me_4_BODIPY-PC mole fraction, were detectable in the emission spectra even though corresponding force-area isotherms indicated no deviation from ideal mixing behavior [11]. The findings suggested that direct monitoring of fluorescence spectral changes could provide a highly sensitive tool for studying lipid lateral interactions in monolayers. However, to extract the information from the multiple emission spectra, that is, as many as 600 spectra per isotherm, an effective means is needed to analyze the data. 

When the input data is extremely large and/or contains redundancy, then transformation into a reduced representation provides an effective means for preservation of essential information. Several data reduction methods are available [13–17] such as principal component analysis (PCA), semidefinite embedding, partial least square, and multidimensional scaling. PCA was designed by Karl Pearson [16] to compress and identify unknown trends in a multidimensional data set. Data dimensionality is reduced by performing a covariance analysis. Herein, we show that PCA provides an effective way to analyze fluorescence spectra of binary constituent lipid monolayers. By using this method, each spectrum can be represented by one point on a plane. We find that points, belonging to the same lipid constituents and experimental conditions, form a curve on the plane where each point belongs to a different mole fraction. The location and shape of the curve are characteristics to the lateral organization of the respective monolayer. Use of phosphatidylcholine (PC) or sphingomyelin (SM), containing either saturated-monounsaturated or disaturated hydrocarbon chains, as the nonfluorescent lipid in the binary constituent monolayers enables evaluation of the role played by matrix lipid phase state and composition in controlling the lateral mixing of the fluorescent lipid by PCA.

## 2. Materials and Methods

1-Palmitoyl-2-oleoyl-*sn*-glycero-3-phosphocholine (POPC) and 1,2-dipalmitoylphosphatidylcholine (DPPC) were purchased from Avanti Polar Lipids (Alabaster AL). Palmitoyl sphingomyelin (PSM) and oleoyl sphingomyelin (OSM) were produced by reacylation of lyso-SM with the desired fatty acyl residue and purified as described previously [18, 19]. 1-Palmitoyl-2-[7-(4,4-difluoro-1,3,5,7-tetramethyl-4-bora-3a, 4a-diaza-*s*-indacene-8-yl)heptanoyl]-*sn*- glycero-3-phosphocholine (Me_4_BODIPY-PC) was synthesized and purified as described earlier [20]. 

### 2.1. Fluorescence Spectra of Binary-Constituent Lipid Monolayers

The Langmuir film balance platform was outfitted for acquisition of fluorescence emission intensity as a function of wavelength while simultaneously measuring surface pressure (*π*) and dipole potential (ΔV) as a function of lipid cross-sectional molecular area (A) of the lipid monolayer [11, 12]. Briefly, BODIPY lipid films were excited at a 90° incident angle using 488 nm unpolarized light from an argon-ion laser (Model 2122-45L, JDS Uniphase, San Jose, CA) equipped with a model-3 light-intensity controller and a fiber optic coupler (Model HPUC-23-488-S-3, FAC-2BL; Oz Optics, Nepean, ON, Canada). Fluorescence emission was collected perpendicular to the interface at a distance of ~1 cm using a fiber optic spectrometer (Model PC2000-ISA, Ocean Optics, Dunedin, FL) equipped with an L2 lens and 200-*μ*m slit. A 500-nm-long pass filter (500EFLP, Omega Optical, Brattleboro, VT) was mounted between the emission collimator and the detector to reduce scattered excitation light. Fluorescence emission spectral intensities were collected each second. Although monolayer compression was continuous during the spectral data acquisition cycle, the fractional change in lipid concentration during each acquisition cycle was ≤0.0073. Control emission spectra were unaffected by gas phase, that is, air or argon, or by 0.01% sodium azide in the subphase buffer.

Subphase buffer was maintained at 24°C via a thermostated, circulating water bath and was produced using water purified by reverse osmosis, activated charcoal adsorption, mixed-bed deionization, then passed through a Milli-Q UV Plus System (Millipore Corp., Bedford, MA), and filtered through a 0.22 *μ*m Millipak 40 membrane. Subphase buffer contained 10 mM potassium phosphate (pH 6.6), 100 mM NaCl, and 0.01% NaN3 and was kept stored under argon, cleaned by passage through a seven-stage series filtration set-up consisting of an Alltech activated charcoal gas purifier, a LabClean filter, and a series of Balston disposable filters consisting of two adsorption (carbon) and three filter units (93% and 99.99% efficiency at 0.1 *μ*m). The film balance was housed in an isolated laboratory supplied with clean air by a Bioclean Air Filtration system equipped with charcoal and HEPA filters and was kept under humidified argon in a separate enclosure. Other features contributing to isotherm reproducibility include automated lipid spreading via a modified HPLC autoinjector, automated surface cleaning by multiple barrier sweeps between runs, and highly accurate, reproducible setting of the subphase level by an automated aspirator. Glassware was acid cleaned, rinsed with purified water, and then with hexane/ethanol (95: 5) before use.

Lipid monolayers were formed by automated spreading (51.67 *μ*L aliquots) of mixtures made from stock solutions dissolved in toluene/ethanol (5:6) or hexane/isopropanol/water (70: 30: 2.5). After spreading on the subphase surface and a delay period of 4 min, lipid films were compressed at a rate of ≤4 Å^2^/molecule/min. Surface pressure and area calibration of the film balance were performed as detailed previously [21]. Phospholipid concentrations, including BPC, were determined by a modified Bartlett assay [21]. Solvent purity was verified by dipole potential measurements using a ^210^Po ionizing electrode [21].

### 2.2. Principal Component Analysis of the Fluorescence Spectra

Here we provide a short description of PCA. The measured fluorescence spectra can be represented by **S**, an *nxm* matrix, where *m* is the number of measured spectra. Each spectrum is measured at the same *n* wavelengths. *S_ij_* is the intensity of fluorescence emission measured at the *i*th wavelength of the *j*th spectrum. The covariance matrix **C**, a symmetric *mxm* matrix, can be created from the **S** matrix as follows:

(1) $$C_{ij}=\frac{\sum_{k=1}^{n}\left(S_{ki}-M_{i}\right)\left(S_{kj}-M_{j}\right)}{n-1},$$

where *M*_*j*_ = ∑_*h*=1_^*n*^ *S*_*hj*_/*n* is the average intensity of the *j*th spectrum. There are *m* eigenvalues and *m* respective eigenvectors for the covariance matrix. The eigenvalues of C are real positive numbers and are sorted in descending order in the eigenvalue vector, Λ. From the eigenvectors, one can create an *mxm* matrix, V where each column represents an eigenvector. In V, the eigenvectors are sorted as in Λ, that is, the eigenvector the eigenvector in the first column has the largest eigenvalue; in the second column, the next largest eigenvalue, and so forth . By multiplying the S and V matrices and transposing the product, one obtains the *mxn* final data matrix, P = (SV)^T^. We refer to the rows of the final data matrix as principal vectors. The *k*th element of the* i*th principal vector is

(2) $$P_{ik}=\overset{m}{\underset{h=1}{\sum }}S_{kh}V_{hi}.$$

In the case of our spectra, the first two eigenvalues in Λ are much larger than the rest of the eigenvalues, as shown in Table 2. Thus each fluorescent spectrum can be approximated by the linear combination of the first two principal vectors, that is

(3) $$S_{kj}\approx M_{j}+V_{j1}P_{1k}+V_{j2}P_{2k},$$

or if the intensity of each spectra at the first wavelength is zero, *S*_1*j*_ = 0, then

(4) $$S_{kj}=S_{kj}-S_{1j}\approx V_{j1}\left(P_{1k}-P_{11}\right)+V_{j2}\left(P_{2k}-P_{21}\right).$$

## 3. Results

**Figure 1:**
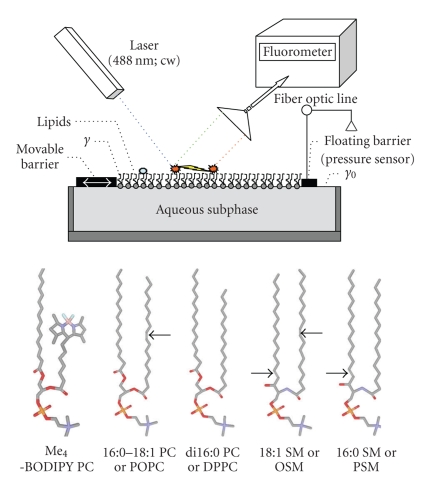
Experimental set-up and structures of lipids investigated. *Upper panel* depicts a Langmuir surface balance modified to acquire fluorescence emission spectra of lipids forming a monolayer at the air/water interface. *γ*_0_ = surface tension of gas/water interface; *γ* = surface tension of gas/water interface covered by lipid; (*γ*_0_-*γ*) = surface pressure. *Lower panel* shows the structures of the lipids studied. The following color scheme applies: gray = carbon, red = oxygen, blue = nitrogen, orange = phosphorus, pink = boron, aqua = fluorine. Arrows pointing left indicate cis double bonds and arrows pointing right equal trans double bonds.

**Figure 2:**
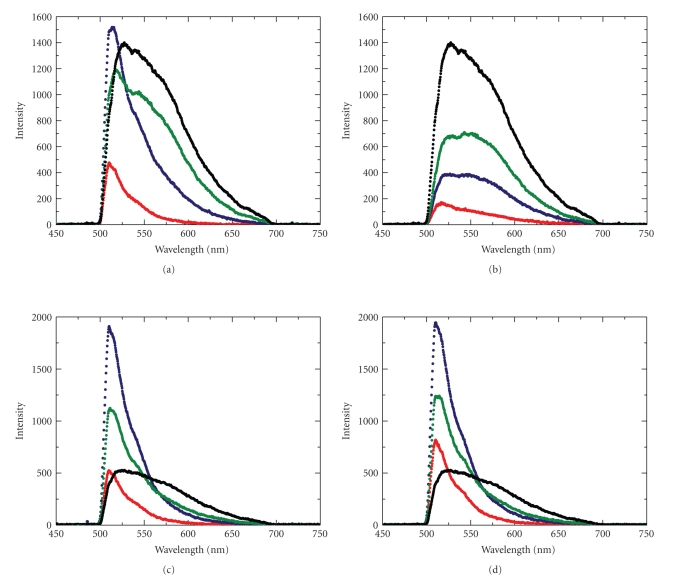
Fluorescence spectra of binary-constituent lipid monolayers containing BODIPY-labeled phosphatidylcholine. Fluorescence emission intensity of BODIPY-labeled PC is plotted against emission wavelength. (a) Me_4_BODIPY-PC/POPC monolayers at 30 mN/m lateral pressure; (b) Me_4_BODIPY-PC/DPPC monolayers at 30 mN/m, (c) Me_4_BODIPY-PC/POPC monolayers at 5 mN/m, (d) Me_4_BODIPY-PC-/DPPC monolayers at 5 mN/m. Color codes: 1% Me_4_BODIPY-PC (red), 10% Me_4_BODIPY-PC (blue), 20% Me_4_BODIPY-PC (green), 100% Me_4_BODIPY-PC (black).

Fluorescence spectra of lipid monolayers containing Me_4_BODIPY-PC as one of the two lipid constituents were acquired while simultaneously measuring the surface pressure versus average molecular area isotherm (Figure 1, upper panel). In every case, Me_4_BODIPY-PC was one lipid constituent (Figure 1, lower panel), while the type and amount of the second lipid constituent (POPC, DPPC, OSM, and PSM) varied. This experimental strategy provided insights into the effects of lipid phase state and composition on Me_4_BODIPY-PC lateral mixing. Figure 2 serves as an example, showing the fluorescence spectra of Me_4_BODIPY-PC/POPC (Figures 2(a) and 2(c)) and Me_4_BODIPY-PC/DPPC (Figures 2(b) and 2(d)) monolayers obtained at two representative surface pressures of 5 and 30 mN/m. It is noteworthy that surface pressures in the 30–35 mN/m region mimic conditions found in biomembranes [22, 23]. For each panel in Figure 2, the four spectra correspond to the following Me_4_BODIPY-PC mole fractions: 0.01 (red), 0.1 (blue), 0.2 (green), 1 (black).

Interpretation of the Me_4_BODIPY-PC spectral responses in Figure 2 is facilitated by consideration of the phase behavior of the lipids comprising the binary-constituent lipid monolayers. POPC and Me_4_BODIPY-PC both are known to display fluid phase behavior at all surface pressures below monolayer collapse (e.g., ~45 mN/m), while DPPC displays fluid phase behavior only at low surface pressures (e.g., <~8 mN/m ) and undergoes a two-dimensional phase transition resulting in ordered lipid hydrocarbon chains at high surface pressures (e.g., [11, 18, 24]). At 5 mN/m (Figures 2(c) and 2(d)), both lipid constituents display fluid-phase behavior and the emission spectra are similar in intensity and shape at equivalent Me_4_BODIPY-PC mole fractions. However, at 30 mN/m (Figures 2(a) and 2(b)), only Me_4_BODIPY-PC and POPC remain fluid; whereas, DPPC is gel-like and chain ordered [11, 18, 24]. Under the latter condition, dramatic changes are observed in Me_4_BODIPY-PC/DPPC spectral intensity and shape, that is, quenching and broadening, compared to equivalent Me_4_BODIPY-PC mole fractions in the Me_4_BODIPY-PC/POPC mixed monolayers.

With BODIPY fluorophores, dilute noninteracting monomers exhibit a narrow emission peak centered in the 506–515 nm range after excitation [11, 12, 25–27]. At sufficiently high BODIPY concentrations, additional absorption and emission peaks are observed, often reflecting dimer emission and/or homo resonance energy transfer (Förster distance = 57 Å). Johansson and colleagues have shown that emission of ground-state dimethyl-BODIPY dimers, denoted D_**II**_ (J-dimer) occurs when the BODIPY rings are oriented in planes with their *S*_0_ → *S*_1_ transition dipoles aligned at ~55°. D_**II**_ dimers absorb near 570 nm and emit near 630 nm [25]. Energy transfer to the ground-state D_**II**_ dimers from excited-state monomers is typically responsible for the D_**II**_ emission peak observed near 630 nm. A second type of ground-state dimer, denoted D_**I**_ (H-dimer) and characterized by sandwich-like stacking of the BODIPY rings, results in parallel alignment of the transition dipoles and absorption near 477 nm, but produces no fluorescence emission after excitation [25, 26]. In the case of Me_4_-BODIPY-PC, we find no evidence of the 620–630 nm peak characteristic of dimethyl-BODIPY dimer fluorescence but rather the presence of an emission shoulder (~570 nm) at high Me_4_BODIPY-PC surface concentrations. Substantial spectral broadening occurs because of increased fluorescence near 570 nm relative to the 510–520 nm region. Thus, detection of surface concentration changes and lateral heterogeneity in the mixing of Me_4_BODIPY-PC in lipid monolayers and bilayers becomes evident.

In addition to formation of the emission shoulder, intensity decreases occur in the ~510 nm emission peaks at 20 mole% compared to 10 mole% Me_4_BODIPY-PC (Figures 2(a), 2(c), and 2(d)). This response could reflect nonfluorescent relaxation occurring for excited monomers by a mechanism involving Förster energy transfer between excited state monomer and ground state dimers aligned differently than D_**II**_ dimers [25, 26]. In contrast to the above cases, the spectra in Figure 2(b) are broad at every Me_4_BODIPY-PC mole fraction, that is, fluorophore dimers form even at very low probe concentration, suggesting highly nonideal mixing and possible lateral phase separation of Me_4_BODIPY-PC in the DPPC monolayers at 30 mN/m (Figure 2(b)). Under such conditions, the specific area of Me_4_BODIPY-PC (30 mN/m; 80 Å^2^/molecule) [11] is considerably larger than the DPPC specific area (46.2 Å^2^/moL) [18, 24]. Me_4_BODIPY-PC molecules are unable to fit into the tight lattice of DPPC molecules. Breaking this lattice structure at many points is not energetically feasible. Thus, even at low concentration, Me_4_BODIPY-PC molecules are expected to segregate, forming clusters in the monolayer. In the clusters, the local probe concentration is high, where dimers (or excimers) may form [25–28], resulting in spectral broadening (Figure 2(b); red curve). The preceding discussion of spectral and force-area data facilitates interpretation of the results of the principal component analyses that follow.

In general, analogous monolayer phase behavior is exhibited by 18:1 sphingomyelin (SM) as by POPC in that 18:1-SM remains fluid at all surface pressures. However, at 30 mN/m, 16:0 SM exists as a mixture of coexisting gel-like chained-ordered and fluid chain-disordered phases, in contrast to DPPC which exists as a gel-like, chain-ordered phase (e.g, [18, 24]). It is noteworthy that the spectra of Me_4_BODIPY-PC/18:1-SM and Me_4_BODIPY-PC/16:0-SM (not shown) are broadened at surface pressures of 5 and 30 mN/m as well as at average molecular cross-sectional areas of 64 Å^2^/molecule and 75 Å^2^/molecule with increasing probe concentration, reflecting subtle differences in lipid structure.

Table 1 shows that a total of 52 fluorescence spectra were simultaneously analyzed by PCA using the spectra of 13 binary mixtures taken at two lateral pressures and two specific areas. We determined the eigenvalues and eigenvectors of the respective 52 × 52 covariance matrix, **C,** by reducing the symmetric matrix to tridiagonal form [16, 17]. The first six elements of the sorted eigenvalue vector, Λ, are shown in Table 2.

**Figure 3:**
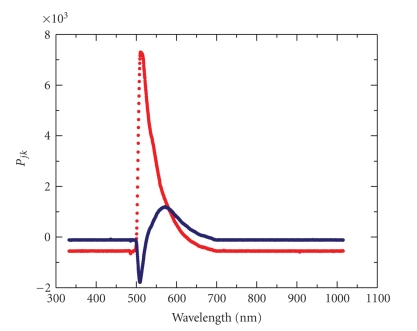
First two principal vectors of 52 fluorescence spectra. The elements of the first two principal vectors, *P*_1*k*_ and *P*_2*k*_, respectively, are plotted against the 2048 wavelengths where each of the 52 fluorescence spectra was measured. Each measured spectrum can be approximated by the linear combination of these two curves (see (4)). The first and second principal vectors are marked by red and blue dots, respectively.

Since the first two eigenvalues are much larger than the rest of the 52 eigenvalues, one is able to characterize each fluorescence spectrum by only the first two principal vectors (first two rows of the final data matrix, **P**). This is a massive dimensionality reduction from 52 to 2. In Figure 3, the components of the first two principal vectors are plotted against the respective wavelengths. The first principal vector (red curve) shows strong similarity to spectra measured at low Me_4_BODIPY-PC mole fractions with the exception of the spectrum (red curve) shown in Figure 2(b). The peak location (510 nm) of the first principal vector and its half width (40 nm) are similar to that of spectra taken at 1 mole% Me_4_BODIPY-PC concentration in Figures 2(a), 2(c), and 2(d). As was pointed out above, these spectra originate from fluorescence emission of the excited monomer probe. The second principal vector (Figure 2, blue curve) resembles higher-order fluorescence, that is, dimer or excimer, because it peaks at 572 nm where the spectral shoulder appears at high Me_4_BODIPY-PC concentrations. Note that each spectrum taken at high Me_4_BODIPY-PC concentration is expected to result in both monomer and dimer (or excimer) fluorescence because dimer formation from monomers is a reversible interaction [25–28].

**Figure 4:**
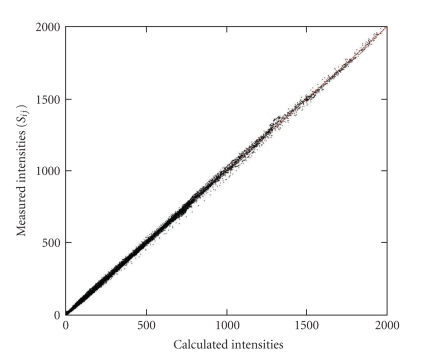
Comparison of reconstructed spectral intensities with experimental intensities. Experimental intensities from 52 fluorescence spectra are plotted against calculated intensities. The calculation is based on an equation, Equation (4), containing the first two principal vectors. The plot contains 52 × 2048 points. Each measured spectra was shifted to get zero average intensity at the first 50 wavelengths.

 In order to demonstrate the validity of the PCA approach, the measured intensities of the 52 fluorescence spectra were plotted against the calculated intensities using (4) (Figure 4). A straight line could be fitted to the 52 × 2048 points with high confidence. The slope and the intercept of the fitted line are 1.0002 ± 0.00008 and −0.043 ± 0.018, respectively, while the correlation coefficient of the fit is 0.9997. Consequently, the spectra of BODIPY-labeled, two-constituent lipid monolayers can be approximated by the linear combinations of the first two principal vectors given by (4). 

**Figure 5:**
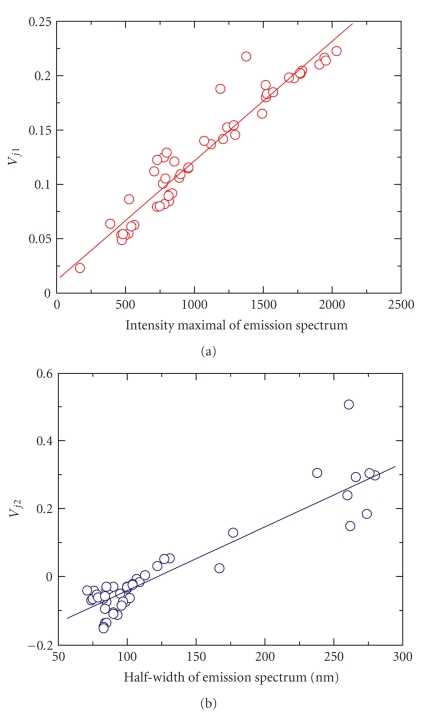
Correlations between eigenvector elements and spectral characteristics. (a) Elements of the first eigenvector of the covariance matrix *V*_*j*1_ are plotted against the peak height of the respective spectrum. The slope and intercept of the fitted straight line are 1.1 · 10^−4^ ± 4.5 · 10^−6^ and 0.012 ± 0.005. The correlation coefficient is 0.96. (b) Elements of the second eigenvector of the correlation matrix *V*_*j*2_ are plotted against the half-width of the respective spectrum. The slope and intercept of the fitted straight line are 0.0019 ± 0.00012 and −0.229 ± 0.017. The correlation coefficient is 0.91.

In the case of the *j*th spectrum, the coefficients of the linear combinations are the *j*th elements of the first two eigenvectors, *V*_*j*1_ and *V*_*j*2_. There is a strong positive correlation between the value of *V*_*j*1_ and the peak height of the *j*th spectrum, while *V*_*j*2_ correlates with the half-width of the spectrum. In Figure 5(a), the elements of the first eigenvector are plotted against the respective peak height. The correlation coefficient between *V*_*j*1_ and the peak height is 0.96. In Figure 5(b), the elements of the second eigenvector are plotted against the half-width of the respective spectrum. The correlation coefficient between *V*_*j*2_ and the half-width is 0.91. Note that in Figure 5(b), the point that deviates most from the general trend, with coordinates (261,0.5), belongs to the spectrum of pure Me_4_BODIPY-PC monolayer at 30 mN/m.

## 4. Discussion

**Figure 6:**
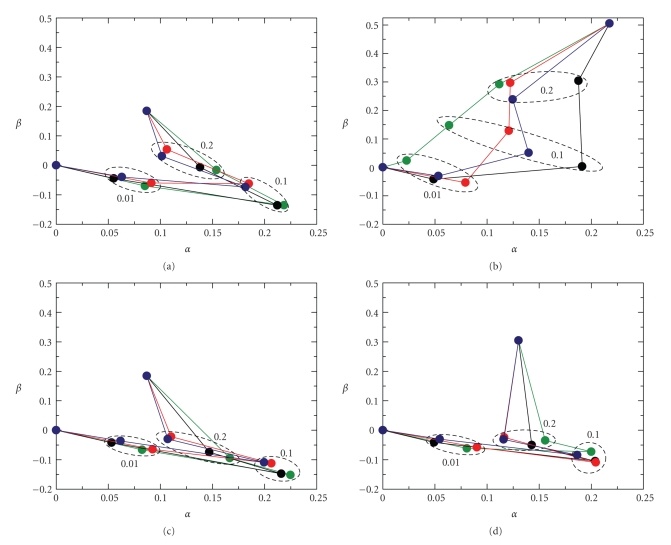
2D-Diagram of fluorescence spectra of binary-constituent lipid monolayers. Each point on the (*α*, *β*) plane represents a fluorescence spectrum of a BODIPY-labeled binary-constituent lipid monolayer. For the *j*th spectrum *α* = *V*_*j*1_ and *β* = *V*_*j*2_, where *V*_*j*1_ and *V*_*j*2_ are *j*th component of the 1st and 2nd eigenvectors, respectively. A zig-zag line of the same color connects points belonging to monolayers comprised of the same lipid constituents. These lines start from the origin representing the spectra of different monolayers with 0 of the monolayer increases with successive dots corresponding to Me_4_BODIPY-PC mole fractions of 0.01, 0.1, and 0.2. All lines converge to the same final point, representing the spectrum of pure Me_4_BODIPY-PC monolayer. Panels (a) and (b) represent lateral pressures of 5 and 30 mN/m; whereas, panels (c) and (d) correspond to average cross-sectional molecular areas of 75 and 64 Å^2^/molecule, respectively. Color codes: Me_4_BODIPY-PC/POPC (black), Me_4_BODIPY-PC/DPPC (green), Me_4_BODIPY-PC/18:1SM (blue), Me_4_BODIPY-PC/16:0SM (red). The dotted ellipses encircle equivalent Me_4_BODIPY-PC mole fractions, with the numerical label indicating the fluorophore mole fraction (0.01, 0.1, or 0.2) mixed with each of the four different nonfluorescent lipids (POPC, DPPC, 18:1-SM, or 16:0-SM).

According to (4), the* j*th elements of the first two eigenvectors, *V*_*j*1_ and *V*_*j*2_, fully specify the* j*th spectrum, while the first two principal vectors are characteristics of all the measured spectra. Thus, geometrically each spectrum can be represented by one point in a plane with coordinate axes: *α* and *β*. For example, in the case of the *j*th spectrum, the coordinates of the respective point are *α* = *V*_*j*1_ and *β* = *V*_*j*2_. Figures 6(a), 6(b), 6(c), and 6(d) show the (*α*, *β*) planes with points representing different spectra, measured at surface pressures of 5 mN/m and 30 mN/m, and at average molecular cross-sectional areas of 75 Å^2^/molecule and 64 Å^2^/molecule, respectively. In each panel, points belonging to monolayers containing the same lipid constituents are connected by the same color line: Me_4_BODIPY-PC/POPC (black); Me_4_BODIPY-PC/DPPC (green); Me_4_BODIPY-PC/18:1-SM (blue); Me_4_BODIPY-PC/16:0-SM (red). The zig-zag nature of the line connecting the points illustrates how the characteristic point representing the spectrum travels as the mole fraction of Me_4_BODIPY-PC increases. When the probe's mole fraction approaches zero, the characteristic point of each line is expected to approach the origin (*α* = 0, *β* = 0). This is the case because, with decreasing Me_4_BODIPY-PC concentration, the fluorescence intensity at any wavelength approaches zero, *S*_*kj*_ → 0, and thus in (4), both *V*_*j*1_ and *V*_*j*2_ should approach zero. (Note that the principal vectors are independent of the Me_4_BODIPY-PC concentration.) Thus in spite of the fact that there is no spectrum at zero fluorophore concentration, each line can be expected to start from the origin. 

It was pointed out above that the *β* and *α* coordinates of the characteristic point correlate with the half-width and height of the spectrum, respectively, and these spectral properties depend on dimer (or excimer) formation by the fluorophore. Since the fluorescence spectrum depends on the surface concentration, that is, mole fraction, of the fluorescent lipid in the respective monolayer, one can expect the location and shape of the lines in the (*α*, *β*) plane to reflect changes in Me_4_BODIPY-PC surface concentration and lateral organization. It is noteworthy that two distinctly different line shapes are observed in Figure 6.

1. The initial slope of the line is negative, starting from zero fluorophore content, if the monolayer is in fluid or fluid/gel mixed phase. This situation occurs for all lipids shown in Figure 6, except for the green curve (DPPC) in Figure 6(b) (30 mN/m). At low-mole fraction, the Me_4_BODIPY-PC is expected to mix ideally with the fluid phase lipids and to be monomerically dispersed. With increasing fluorophore concentration, the number of monomers increases (i.e., the peak height and *α* increases), and the half-width of the spectrum slightly decreases (i.e., *β* decreases). Between 10 and 20 mole% Me_4_BODIPY-PC, dimers are expected to substantially increase in the monolayer, resulting in an emission shoulder at 570 nm (i.e., the half-width and *β* increases). On the other hand, dimer formation makes possible nonfluorescent energy transfer between excited state monomer and ground state dimer (i.e., the peak height and *α* decreases).
2. The initial slope of the line is positive, starting from zero fluorophore content, if the monolayer is in gel phase (Figure 6(b), green curve). Even at low-mole fraction, Me_4_BODIPY-PC mixes nonideally with gel phase DPPC, and forms aggregates. Thus increasing monomer and dimer fluorescence can be detected simultaneously from low-fluorophore concentration, that is, *α* and *β* increase simultaneously with increasing peak height and half-width. The response suggests that dimer abundance can be affected by the lipid lateral organizational state.

## 5. Conclusion

Under fluid-phase monolayer conditions, curves are highly similar for same-type lipids (e.g., POPC and DPPC; OSM and PSM). Nonetheless, detectable differences exist between the lipid types, consistent with subtle differences in the lateral mixing of Me_4_BODIPY-PC in each lipid type. The differences in curves generated from spectra acquired at 30 mN/m imply that both lipid type and phase state affect Me_4_BODIPY-PC lateral distribution under lipid packing conditions approximating biomembranes. Currently underway are comprehensive studies involving: (1) more lipid mixing ratios, (2) different BODIPY-labeled lipids, and (3) other nonfluorescent lipid components.

Collectively, our data demonstrate the usefulness of PCA for deciphering fluorescence spectra obtained from lipid monolayers with respect to the lateral distribution of lipid fluorophore. The exceptional promise shown by PCA for handling large spectral data sets, the nanoscale resolution afforded by the fluorescence signal, and the inherent versatility of lipid monolayers for characterization of lipid lateral interactions combine to provide a new approach expected to facilitate investigation of lipid lateral organizational changes induced by differing lipid compositions and by proteins.

## Figures and Tables

**Table 1:** Compositions and experimental conditions of binary-constituent lipid monolayers.

	5 mN/m	75 Å^2^/molecule	30 mN/m	64 Å^2^/molecule
Me_4_BODIPY-PC/POPC	1%	1%	1%	1%
Me_4_BODIPY-PC/POPC	10%	10%	10%	10%
Me_4_BODIPY-PC/POPC	20%	20%	20%	20%
Me_4_BODIPY-PC/DPPC	1%	1%	1%	1%
Me_4_BODIPY-PC/DPPC	10%	10%	10%	10%
Me_4_BODIPY-PC/DPPC	20%	20%	20%	20%
Me_4_BODIPY-PC/18:1-SM	1%	1%	1%	1%
Me_4_BODIPY-PC/18:1-SM	10%	10%	10%	10%
Me_4_BODIPY-PC/18:1-SM	20%	20%	20%	20%
Me_4_BODIPY-PC/16:0-SM	1%	1%	1%	1%
Me_4_BODIPY-PC/16:0-SM	10%	10%	10%	10%
Me_4_BODIPY-PC/16:0-SM	20%	20%	20%	20%
Me_4_BODIPY-PC	100%	100%	100%	100%

% indicates Me_4_BODIPY-PC mole%.

The first and third columns represent surface pressure.

The second and fourth columns represent cross-sectional molecular area.

**Table 2:** First six eigenvalues of the covariance matrix.

*λ*_1_	*λ*_2_	*λ*_3_	*λ*_4_	*λ*_5_	*λ*_6_
2,264,835	158,654	594	316	192	52
